# Altered Hippocampal Clock Gene Regulation Is Associated with Circadian Dysregulation of Oxidative Imbalance, Neuroinflammation, and Histopathological Damage After Pinealectomy

**DOI:** 10.3390/biology15080655

**Published:** 2026-04-21

**Authors:** Venhar Gurbuz Can, Mehmet Demir, Tansu Kusat, Feyza Basak

**Affiliations:** 1 Department of Medical Biology, Faculty of Medicine, Karabuk University, Karabuk 78050, Turkey; 2 Department of Physiology, Faculty of Medicine, Karabuk University, Karabuk 78050, Turkey; mehmetdemir@karabuk.edu.tr; 3 Department of Histology and Embryology, Faculty of Medicine, Karabuk University, Karabuk 78050, Turkey; tansukusat@karabuk.edu.tr (T.K.); feyzabasak@karabuk.edu.tr (F.B.)

**Keywords:** clock genes, immunohistochemistry, neuroinflammation, oxidative stress, pinealectomy

## Abstract

Pinealectomy leads to melatonin deficiency, which is known to disrupt circadian clock regulation and may increase vulnerability of the hippocampus to oxidative stress and neuroinflammatory processes. The objective of this study was to examine the gene expression levels of circadian locomotor output cycles kaput (CLOCK), brain and muscle ARNT-like 1 (BMAL1), period circadian regulator 1 (PER1), cryptochrome circadian regulator 1 (CRY1), brain-derived neurotrophic factor (BDNF), and interleukin-6 (IL-6) in the hippocampus to elucidate the impact of pinealectomy-induced circadian dysregulation on these gene expressions and to assess its association with hippocampal alterations. A total of 30 Wistar rats were randomly divided into three groups: Control, Sham, and Pinealectomy (PNX) (*n* = 10 per group). Gene expression levels were analyzed using quantitative real-time polymerase chain reaction (qRT-PCR). Immunohistochemical analysis was performed to assess caspase-3 and glial fibrillary acidic protein (GFAP) immunoreactivity. In addition, oxidative stress parameters, including malondialdehyde (MDA), superoxide dismutase (SOD), catalase (CAT), and glutathione (GSH), as well as the inflammatory marker tumor necrosis factor-alpha (TNF-α), were measured. The pinealectomy group showed a significant downregulation of BMAL1, BDNF, CLOCK, CRY1, and PER1 gene expression levels, with decreases ranging from approximately 60% to 83% compared with the sham and control groups, whereas IL-6 expression was significantly increased by approximately 185% (*p* < 0.05). Immunohistochemical analysis demonstrated significantly increased caspase-3 and GFAP immunoreactivity in the PNX group. Furthermore, pinealectomy resulted in a significant increase in MDA and TNF-α levels, accompanied by marked decreases in SOD, CAT, and GSH levels (*p* < 0.05). In conclusion, pinealectomy is associated with significant disruption of hippocampal circadian clock gene expression, accompanied by oxidative stress, neuroinflammation, and histopathological alterations. These findings highlight the critical role of circadian regulation in maintaining hippocampal cellular integrity.

## Simple Summary

The present study examined the impact of pineal gland removal on the hippocampus and found that pinealectomy disrupted genes involved in biological timing and was associated with increased oxidative stress, reduced antioxidant defence, enhanced neuroinflammation, and decreased levels of a brain-supporting factor involved in neuronal survival. These molecular changes were accompanied by structural damage in the hippocampus, including increased cell death and astrocyte activation, which play important roles in maintaining neuronal structure and function. The present study suggests that disruption of normal circadian regulation may increase the hippocampus’s vulnerability to inflammation and oxidative damage.

## 1. Introduction

Circadian rhythms play a key role in regulating physiological [1] and pathological responses to daily environmental changes [2]. The circadian system consists of three main components: the clock, the retinohypothalamic tract, and melatonin, which is secreted by the pineal gland and acts as a marker for the clock [3]. The pineal gland, located in the epithalamus, is an important component of circadian mechanisms [4] and is closely linked to the light–dark phases of the daily cycle, exhibiting neuroendocrine functions [5]. The synthesis of melatonin, which occurs as a consequence of the stimulation of the pineal gland, is subject to the regulation of the circadian clock located within the suprachiasmatic nucleus (SCN) of the hypothalamus [4,6]. The timepiece in question is synchronised with the light/dark cycle through exposure to retinal light [7]. The synthesis and release of melatonin, a hormone secreted during nocturnal hours, are regulated by circadian rhythms and light signals [8]. Melatonin has been detected in all biological fluids, including cerebrospinal fluid, saliva, bile, joint fluid, amniotic fluid, and breast milk [9,10]. The compound’s hydrophobic nature enables passive diffusion across membranes and the blood–brain barrier [11,12]. Melatonin has been demonstrated to exert several biological effects, including antioxidant, anti-inflammatory, and cytokine-stimulating effects [13]. However, removing the pineal gland (pinealectomy) leads to the rapid accumulation of oxidatively damaged products due to decreased melatonin levels, which act as free radical scavengers and antioxidants [14]. It is suggested that the presence or supplementation of melatonin restores the circadian rhythm by mitigating the effects of genetic variation in the BMAL1 and PER1 clock genes [15]. Research has indicated that the circadian clock system predominantly comprises a series of clock genes, and that the cyclical expression of numerous genes plays a regulatory role in the molecular mechanism of the circadian clock [16,17]. The clock genes have been shown to synchronize behavioral and biochemical processes with the night/day cycle [18]. In mammals, interactions among CLOCK, neuronal PAS domain protein 2 (NPAS2), BMAL1, period proteins (PER1, PER2, and PER3), and cryptochrome proteins (CRY1 and CRY2) are fundamental to the generation of circadian oscillations. BMAL1 and CLOCK proteins are helical–loop–helical (bHLH)-Per-Arnt-Sim (PAS) transcription factors that form heterodimer complexes via Per-Arnt-Sim (PAS) domains [19]. The PER and CRY proteins have been observed to translocate to the nucleus, forming the core negative arm of the transcriptional/translational feedback loop. This process involves inhibiting the CLOCK and BMAL1 genes, thus regulating their transcription [20]. In addition to this direct transcriptional feedback, the mRNA expression of the PER1, PER2, PER3, CRY1 and CRY2 genes is also regulated by various mechanisms [21].

BDNF is a neurotrophic factor that facilitates the growth, development, and survival of neurons in the nervous system. Significant brain regions, including the hippocampus, have been shown to express BDNF protein and mRNA, with changes in BDNF levels linked to neurodegenerative diseases affecting the hippocampus and parahippocampal regions [22,23]. Pinealectomy has been proposed to exacerbate tissue damage caused by reactive oxygen species [24]. Furthermore, interleukin-6 (IL-6) is a multifunctional cytokine that plays a role in immune control and energy metabolism. The effect of IL-6 is further complicated by sex-specific changes in immune function, circadian rhythm, and metabolism [25]. Some studies have shown that pinealectomy reduces BDNF expression [26] and increases the levels of certain pro-inflammatory [27]. Therefore, we hypothesized that the inflammatory process and BDNF expression should also be evaluated to fully elucidate the relationship between pinealectomy and clock genes.

Clock genes are known for regulating critical biological processes, such as cell proliferation, the cell cycle, and apoptosis [28]. Moreover, the rhythmic metabolic function of astrocytes has been identified as essential for circadian regulation in the SCN. However, our comprehension of the relationship between the circadian clock and certain astrocyte functions remains inadequate [29]. Melatonin demonstrates neuroprotective effects by preventing apoptosis in multiple models of central nervous system injury [30]. On the other hand, astrocytes, the predominant glial cells in cerebral tissue, may serve as targets for melatonin [30]. Based on this information, we performed immunohistochemical experiments following pinealectomy to correlate clock gene expression in the hippocampus with apoptotic processes and alterations in GFAP expression in astrocytes.

SOD and CAT are essential antioxidant enzymes against oxidative stress [31]. GSH [32], an intracellular antioxidant, protects neurons and glial cells from oxidative stress [33]. Oxidative stress increases with GSH reduction [34,35]. MDA, a lipid peroxidation end product, is another indicator of oxidative stress [36]. Research shows that pro-inflammatory cytokines and chemokines contribute to neurological and neurodegenerative disorders [37]. Pinealectomy surgery has been reported to negatively affect oxidative stress and inflammation markers in brain tissue [38].

This study aimed to investigate the genes CLOCK, BMAL1, PER1, and CRY1 in the hippocampus post-pinealectomy, along with the genes BDNF and IL-6, which were hypothesized to be affected, using quantitative qRT-PCR. This study elucidates the histological consequences of pinealectomy on hippocampal microstructure and alterations in immunoreactivity, using antibodies against caspase-3 and GFAP, thereby revealing their interconnections. Oxidative stress and inflammatory markers were also included to provide a comprehensive evaluation of the effects of pinealectomy on the hippocampus.

## 2. Materials and Methods

### 2.1. Ethical Approval

Ethics committee approval for the study was obtained from Karabük University Animal Experiments Local Ethics Committee (10 March 2025, E-55212866-050.04-423367). All the authors indicate that all animal experiments comply with the ARRIVE guidelines and are carried out following the U.K. Animals (Scientific Procedures) Act, 1986, and associated guidelines, EU Directive 2010/63/EU for animal experiments, or the National Institutes of Health guide for the care and use of Laboratory animals (NIH Publications No. 8023, revised 1978).

### 2.2. Animals and Conditions

**Figure 1:**
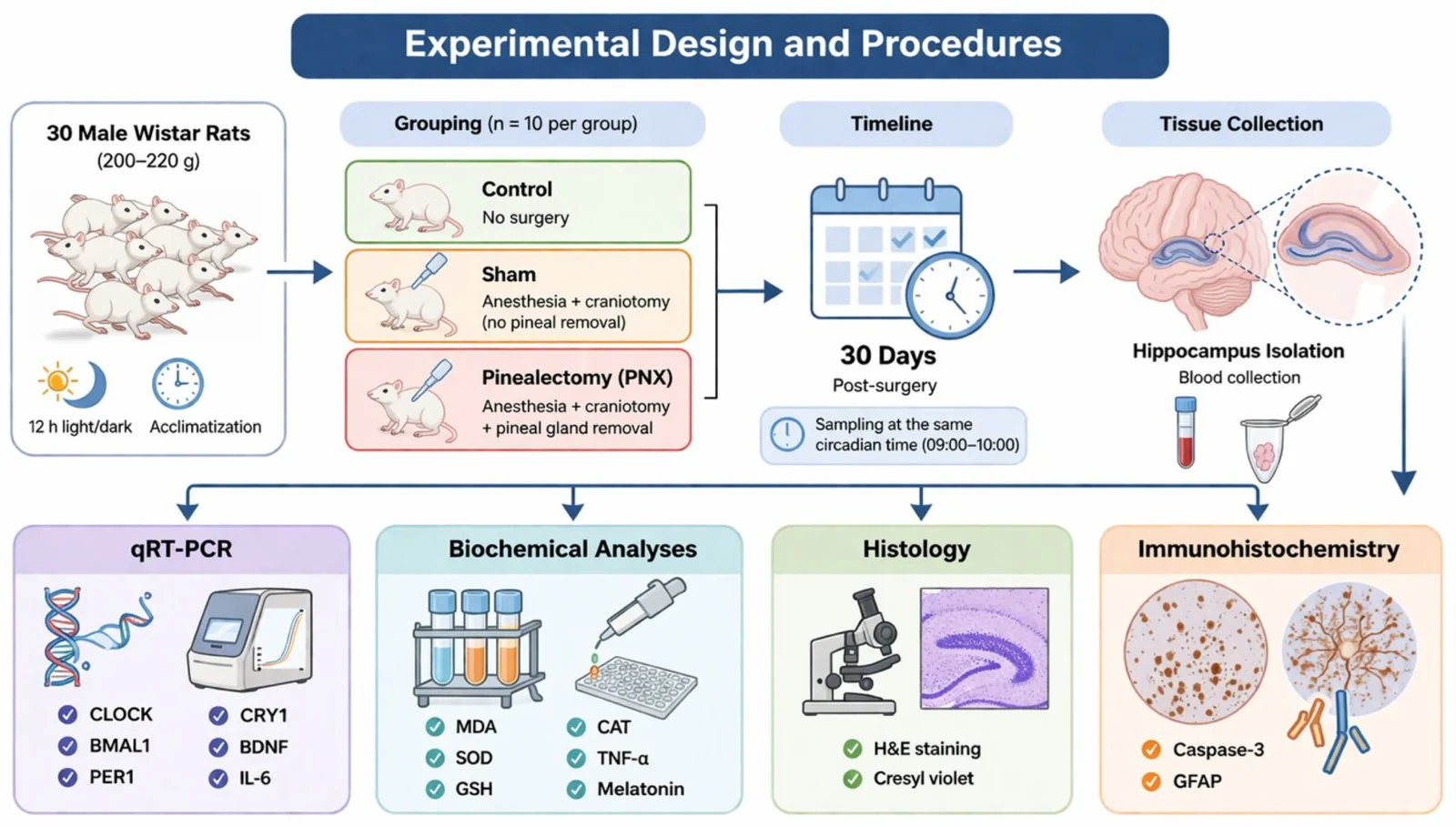
Summary of the experimental design and workflow.

In this study, 30 adult male Wistar albino rats weighing at least 200 g were utilized. The rats were procured from the Experimental Medicine Research and Application Center (DETUM) at Karabük University. During the experiments, the animals were housed in an environment maintained at 21 ± 1 °C and subjected to a 12-h light/dark cycle. They were provided unrestricted access to standard tap water and commercially available rat food. Male rats were used to avoid variability associated with hormonal fluctuations of the estrous cycle, which may influence circadian regulation, oxidative stress, and neuroinflammatory responses [39,40,41]. Anesthesia was induced in rats using a xylazine–ketamine mixture (8 mg/kg + 80 mg/kg, respectively) [42]. Although animals had ad libitum access to food and water, all experimental procedures were performed at a consistent time point to minimize potential feeding-related circadian variability. The following roadmap illustrates the structure of our study (Figure 1).

### 2.3. Experimental Groups

The experimental groups were designed as three groups. Each group comprised ten rats, selected at random. Control Group: No surgical procedures were conducted on the rats within this group. Sham Group: A sham procedure was performed on the rats in this group. All surgical procedures were performed using the established protocols outlined in the section dedicated to pinealectomy surgery in the sham procedure. However, the surgical site was closed without removing the pineal gland. Pinealectomy Group: Rats in this group underwent a surgical procedure known as a pinealectomy.

### 2.4. Pinealectomy Surgery

Rats were sedated using a mixture of xylazine (8 mg/kg) and ketamine (80 mg/kg) delivered via intraperitoneal injection. After anesthesia, rats were positioned in a stereotaxic apparatus, and a midline incision was executed in the skin between the posterior aspect of the snout and the nape of the neck, equidistant from both ocular regions. The periosteum was removed with a scalpel to expose the lambda. A circular incision was made in the upper region of the skull with a micro drill (Proxxon Micromot 50/E, part number 28510, in Wecner, Luxembourg), and the pineal gland was excised with forceps. Following the repositioning of the excised bone fragment, the skin was sutured, and a dressing was placed. Thirty days post-pinealectomy, blood samples were taken from the heart, rats were decapitated under anesthesia, and the hippocampus was subsequently excised [43]. All animals were sacrificed between 09:00 and 10:00 a.m. to minimize the potential effects of circadian variation on gene expression and biochemical parameters [44].

### 2.5. Total RNA Extraction

Following the tissue homogenisation, total RNA was extracted from the samples using the High Pure RNA Isolation Kit (Cat. No.: 11 828 665 001, Roche, Indianapolis, IN, USA). The extraction process was carried out according to the kit manufacturer’s instructions, and a volume of 30–50 μL of total RNA was obtained. Total RNA was stored at −80 °C until the study was conducted.

### 2.6. cDNA Analysis

RNA concentrations were measured using a spectrophotometer (Colibri Titertek Berthold, Pforzheim, Germany) and normalized before cDNA synthesis. The quality and purity of RNA samples were assessed spectrophotometrically. All samples exhibited A260/280 and A260/230 ratios of approximately 2.0, indicating high purity and suitability for RT-qPCR analysis. Subsequently, cDNA synthesis was performed using the OneScript^®^ Plus cDNA Synthesis Kit (ABM Cat. G236, Richmond, BC, Canada) according to the manufacturer’s guidelines, yielding a final reaction volume of 20 μL. After preparing the reaction mixture, the samples were loaded into a conventional PCR machine (Thermo Fisher Scientific Veriti, Waltham, MA, USA). The protocol consisted of one transcription cycle at 25 °C for 10 min and one at 50 °C for 30 min. After the reaction was completed, the enzyme was inactivated at 85 °C for 5 min. After inactivation, the cDNA products were stored at −20 °C.

### 2.7. Quantitative Real-Time PCR Analysis

The expression of the β-actin (ACTB), CLOCK, BMAL1, PER1, CRY1, BDNF, and IL-6 genes was analysed by qRT-PCR. The housekeeping gene ACTB was utilised as a normaliser in the study (see Table 1 for details). ACTB was used as the reference gene for normalization, as it is commonly applied in hippocampal gene expression studies. No marked variation in ACTB Ct values was observed among experimental groups. In the present study, β-actin (ACTB) was used as the reference gene, and its stability was evaluated across the experimental groups (PNX, Sham, and Control). The Ct values of β-actin showed a narrow distribution range across all samples (24.62–26.60). The mean Ct values were very similar between groups (PNX: 25.6, Sham: 25.4, Control: 25.5), and no statistically significant differences were observed (*p* > 0.05). In addition, the standard deviation of Ct values was low (approximately 0.5 Ct), indicating stable gene expression.

The Blastaq™ 2×qPCR MasterMix-ROX (ABM Cat. No. G891, Richmond, BC, Canada) was utilised to ascertain the levels of gene expression. The total volume for the 1X PCR reaction was 20 μL, consisting of 10 μL of master mix, 5 μL of mixB (containing 0.3 μM forward and reverse primers, see Table 1) and 5 μL of cDNA. The reaction was performed on an RT-PCR instrument (BioRAD CFX-96, Hercules, CA, USA) using 0.1 mL microcentrifuge tubes. The temperature was initially increased to 95 °C for 5 min, followed by 40 cycles of 95 °C for 5 s (denaturation), 59 °C for 30 s, 72 °C for 5 s, and 95 °C for 5 s, 60 °C for 1 min, and HRM analysis up to 95 °C. The relative mRNA expression levels obtained for specific genes were determined by means of the 2^−ΔΔCt^ method. qRT-PCR measurements were performed in technical triplicate.

### 2.8. Hematoxylin Eosin Staining

Hippocampus tissues preserved in buffered formalin solution were washed overnight with running water, followed by dehydration in graded alcohols, clearing and polishing in xylene, and embedding in paraffin to produce blocks. Hematoxylin and eosin staining was conducted to elucidate the overall morphology of the hippocampus tissue. After xylol treatment and a graded alcohol series, sections were stained with Hematoxylin (MERCK^®^, 1.04302.0100) for 5 min and then rinsed. Thereafter, they were conserved in acid alcohol and ammonia solutions. Subsequently, the slices were stained with eosin (MERCK^®^, 1.15935.0025) for 2 min. After dehydration and xylene treatment, the slides were affixed with Entellan^®^ (MERCK, Darmstadt, Germany).

### 2.9. Cresyl-Violet Staining

After tissue sectioning, the hippocampal sections were dewaxed and rehydrated in alcohol. The sections were then stained with 0.1% Cresyl Violet (MERCK^®^, 115940) for 10 min. Following dehydration and xylene treatment, the slides were mounted using Entellan^®^.

### 2.10. Immunohistochemistry Staining

Immunohistochemical staining was performed on 4-μm-thick sections obtained from the blocks mounted on adhesive slides. The hippocampus sections were dewaxed and rehydrated in alcohol. To reveal antigenic structures (antigen retrieval), sections in citrate buffer (pH: 6) were boiled in a microwave oven for 15 min. Sections were subjected to 3% hydrogen peroxide for 30 min to suppress endogenous peroxidase activity. Nonspecific binding was inhibited using Protein Block Solution for 5 min at ambient temperature. The sections were incubated overnight at 4 °C in a dark, humid atmosphere with the primary antibody, anti-caspase-3 (Santa Cruz Biotechnology, Dallas, TX, USA; sc-56053) and GFAP (Santa Cruz Biotechnology, Dallas, TX, USA; sc-33673) with a dilution of 1:50. The following day, the antibodies underwent a second wash with PBS, followed by the addition of a biotinylated secondary antibody, which was incubated for 20 min. Subsequently, the sample was washed with PBS and incubated with the enzyme conjugate for 20 min. The tissue slices, previously rinsed with PBS, were stained using a dye formulated with DAB chromogen and DAB substrate (staining was conducted under supervision for a maximum of 5 min). Mayer’s hematoxylin was employed for background staining. The staining was examined using an Olympus BX50 light microscope (Olympus Corporation, Tokyo, Japan) and the attached camera system. Following dehydration and xylene treatment, the slides were mounted using Entellan^®^.

### 2.11. Immunohistochemical Evaluation Procedure

The H-Score values were calculated by counting positively stained cells in 10 randomly chosen fields for each group. The staining intensity in the cell was classified as 0 (negative), 1 (weak), 2 (intermediate), or 3 (strong). The total cell count and their intensity in each field were documented. The following formula was employed: H-score: (% of cells stained at intensity category 1 × 1) + (% of cells stained at intensity category 2 × 2) + (% of cells stained at intensity category 3 × 3). Immunostained slides were evaluated utilizing a research microscope (Leica DM2500 LED, Leica Microsystems, Wetzlar, Germany) equipped with an MC170 HD camera adapter (Leica Microsystems, Wetzlar, Germany). All histopathological and immunohistochemical evaluations were performed by a blinded observer who was unaware of the experimental groups.

### 2.12. Biochemical Analysis

The tissues were retrieved from the freezer and promptly weighed to prepare the tissue homogenate. The tissues were homogenized on ice with a suitable phosphate buffer using a homogenizer to generate a homogenate. After utilizing the homogenate for MDA analysis, the supernatant was isolated from tissue debris by centrifuging the homogenate at 3500 rpm for 15 min at 4 °C to conduct additional biochemical experiments. The assays for MDA and GSH were conducted colorimetrically utilizing the methodology of Ohkawa et al. [45]. The levels of antioxidant enzymes in the tissues were assessed by measuring the activities of SOD and CAT, following the methodologies established by Sun et al. [46] and Aebi [47], respectively. The protein concentration in brain tissues was assessed using the Lowry method [48]. The results of MDA and GSH levels were expressed as nmol/g wet tissue, SOD activity as U/g protein, and CAT activity as K/g protein. The commercial rat ELISA kits (Catalog Number: E0764Ra, Bioassay Technology Laboratory, Shanghai, China) were utilized to quantify the levels of inflammatory cytokines in tissues. TNF-α assessment was conducted according to the manufacturer’s protocols. Serum melatonin levels were measured using the designated rat kits (Cat. No.: E0601Ra, Bioassay Technology Laboratory). The serum analyses were conducted according to the manufacturer’s guidelines. The results are presented in ng/L.

### 2.13. Statistical Analysis

All statistical analyses were performed using GraphPad Prism (Version 8.02; GraphPad Software, San Diego, CA, USA) software. The data presented herein were obtained from a minimum of three independent experiments and are expressed as the mean ± standard deviation (SD). All measurements were performed on samples obtained from *n* = 10 animals per group. Each assay was conducted in technical triplicate where applicable. The data were found to be normally distributed, as indicated by the Kolmogorov–Smirnov test (*p* > 0.05). To assess statistical differences among the experimental groups (Control, Sham, and PNX) gene expression, histological, and biochemical data were analyzed separately for each parameter using one-way analysis of variance (ANOVA). Subsequently, differences between groups were analyzed using Tukey’s multiple comparison post hoc test. Statistically, a value of *p* < 0.05 was considered to be significant.

## 3. Results

### 3.1. qRT-PCR Results

**Figure 2:**
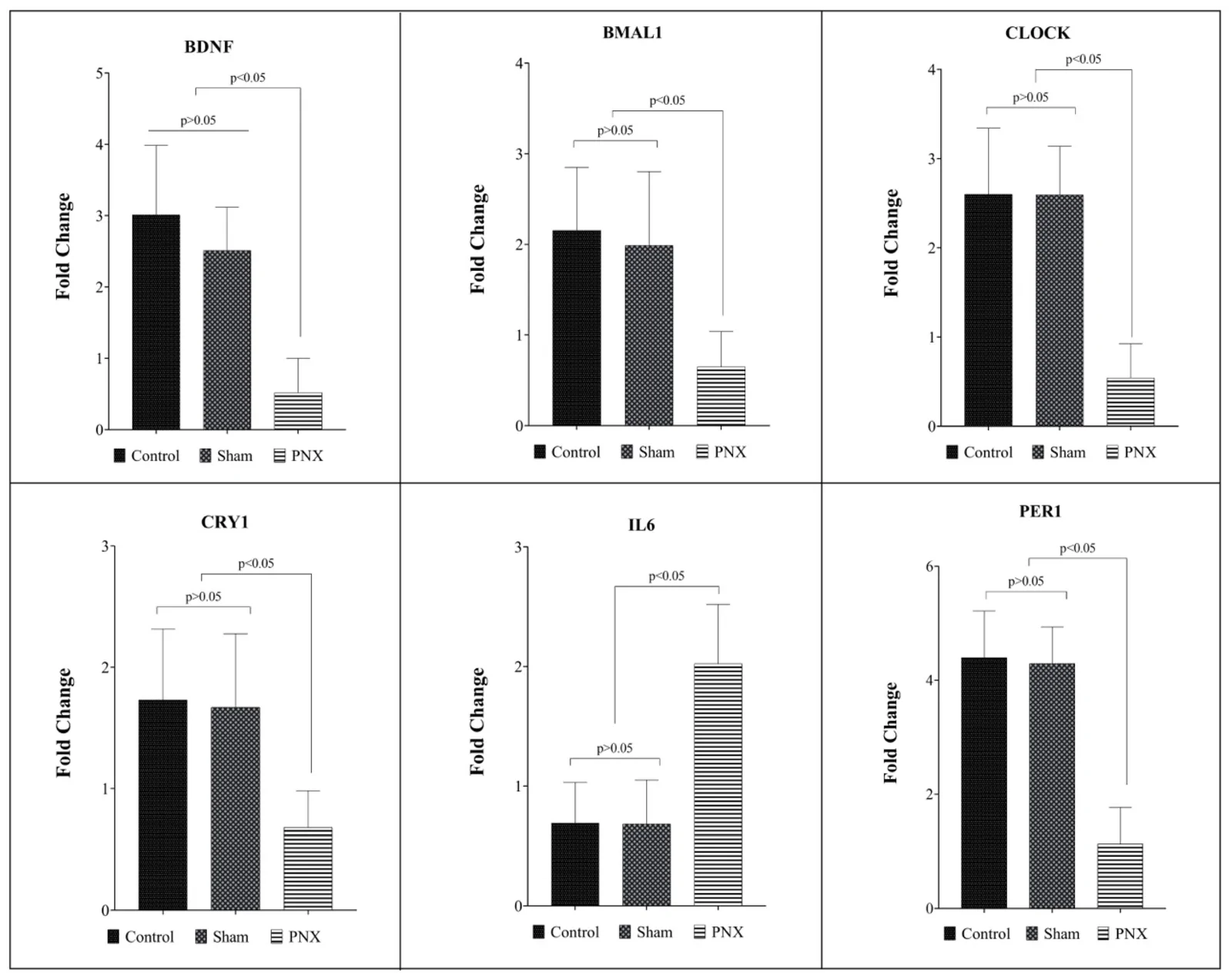
Gene expression levels are presented as mean ± SD (*n* = 10). Significant differences were observed between PNX and both control and sham groups (*p* < 0.05). No differences were observed between the control and sham groups.

Figure 2 illustrates the relative mRNA expression levels of BMAL1, CLOCK, CRY1, PER1, BDNF, and IL-6 genes in the hippocampus of control, sham, and pinealectomized (PNX) rats. The results demonstrated that the expression levels of BMAL1 (0.649 ± 0.389, *p* = 0.002), CLOCK (0.541 ± 0.384, *p* < 0.001), CRY1 (0.680 ± 0.302, *p* = 0.005), PER1 (1.134 ± 0.634, *p* < 0.001), and BDNF (0.519 ± 0.481, *p* < 0.001) were significantly decreased in the PNX group compared with both the control (BMAL1: 2.154 ± 1.229; CLOCK: 2.500 ± 1.117; CRY1: 1.730 ± 0.878; PER1: 4.397 ± 1.393; BDNF: 3.146 ± 0.963) and sham groups (BMAL1: 1.988 ± 0.929; CLOCK: 2.692 ± 1.056; CRY1: 1.669 ± 0.877; PER1: 4.393 ± 1.556; BDNF: 2.512 ± 0.608). In contrast, IL-6 gene expression was significantly increased in the PNX group (2.023 ± 0.955, *p* < 0.001) compared with the control (0.690 ± 0.341) and sham groups (0.683 ± 0.368). No statistically significant differences were observed between the control and sham groups for any of the analyzed genes (*p* > 0.05), indicating that the surgical procedure alone did not affect hippocampal gene expression. Overall, pinealectomy was associated with significantly reduced hippocampal expression of BMAL1, CLOCK, CRY1, PER1, and BDNF, along with increased IL-6 expression, compared with control and sham groups (*p* < 0.05).

### 3.2. Results of Histopathology and Immunohistochemistry

**Figure 3:**
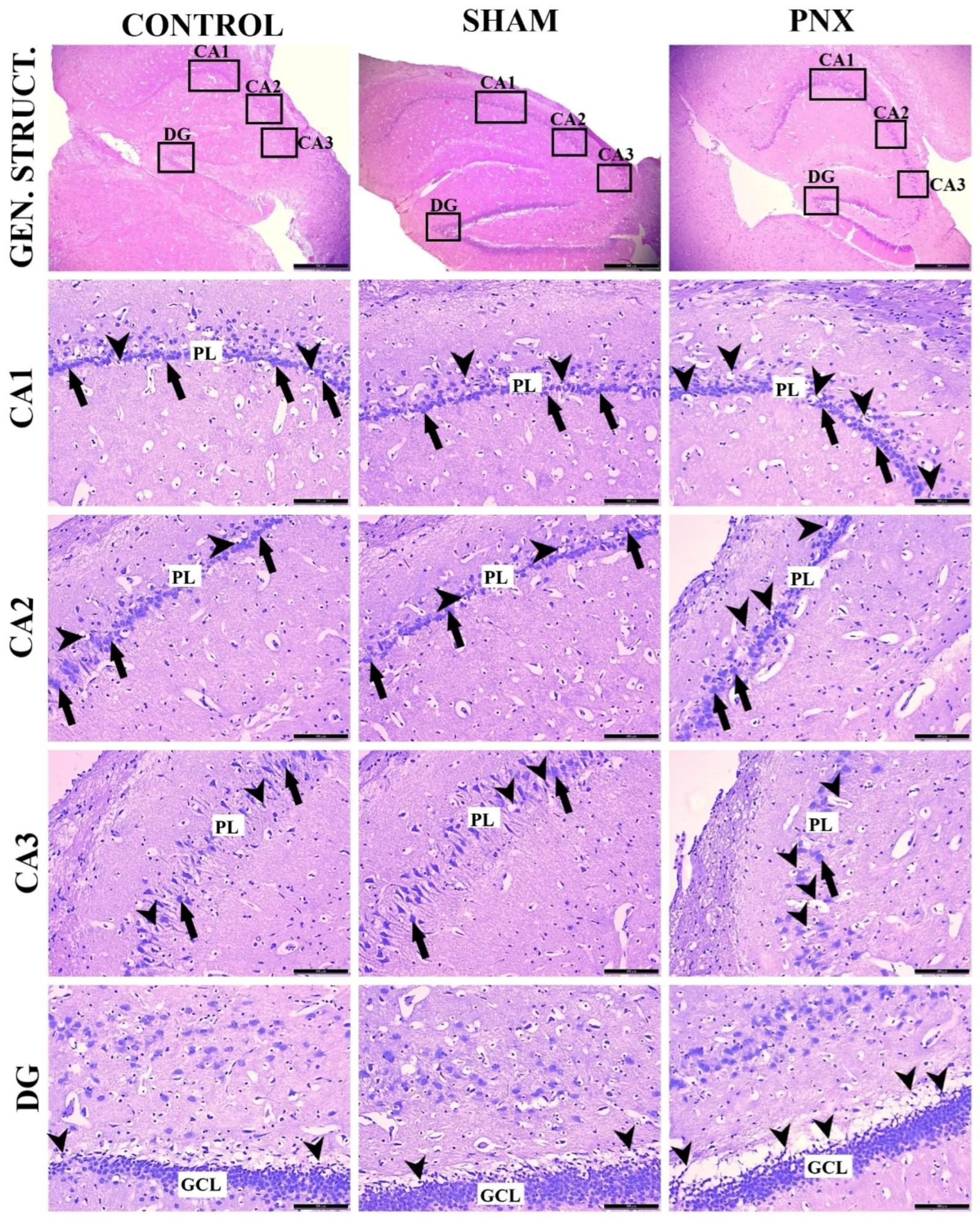
GEN. STRUCT.: General Structure; CA1: cornu ammonis 1; CA2: cornu ammonis 2; CA3: cornu ammonis 3; DG: dentate gyrus; PNX: pinealectomy group. PL: pyramidal layer; GCL: granular cell layer; arrow: neurons presenting normal histological appearance; arrow head: darkly stained cells exhibiting shrinkage of cell bodies, pyknotic nuclei, and pericellular halos. For the General structure micrographs—Hematoxylin Eosin stain, Bar: 500 μm. For CA1, CA2, CA3 and DG micrographs—Cresyl-Violet stain, the bars present 100 μm.

Histological examination revealed the hippocampus as a structure extending toward the base of the temporal horn of the lateral ventricle. The histological evaluation of the hippocampus revealed a normal histological structure without any detectable pathological changes in the control and sham groups. In contrast, the PNX group demonstrated histopathological alterations in the subregions of the hippocampus, including the cornu ammonis regions (CA1, CA2, CA3) and the dentate gyrus. Microscopic evaluation suggested features consistent with neuronal degeneration, including atrophied pyramidal and granule cells, pericellular halos, and intensely stained nuclei with apparent nucleolar depletion (Figure 3).

#### 3.2.1. Caspase-3 Immunohistochemical Staining

**Figure 4:**
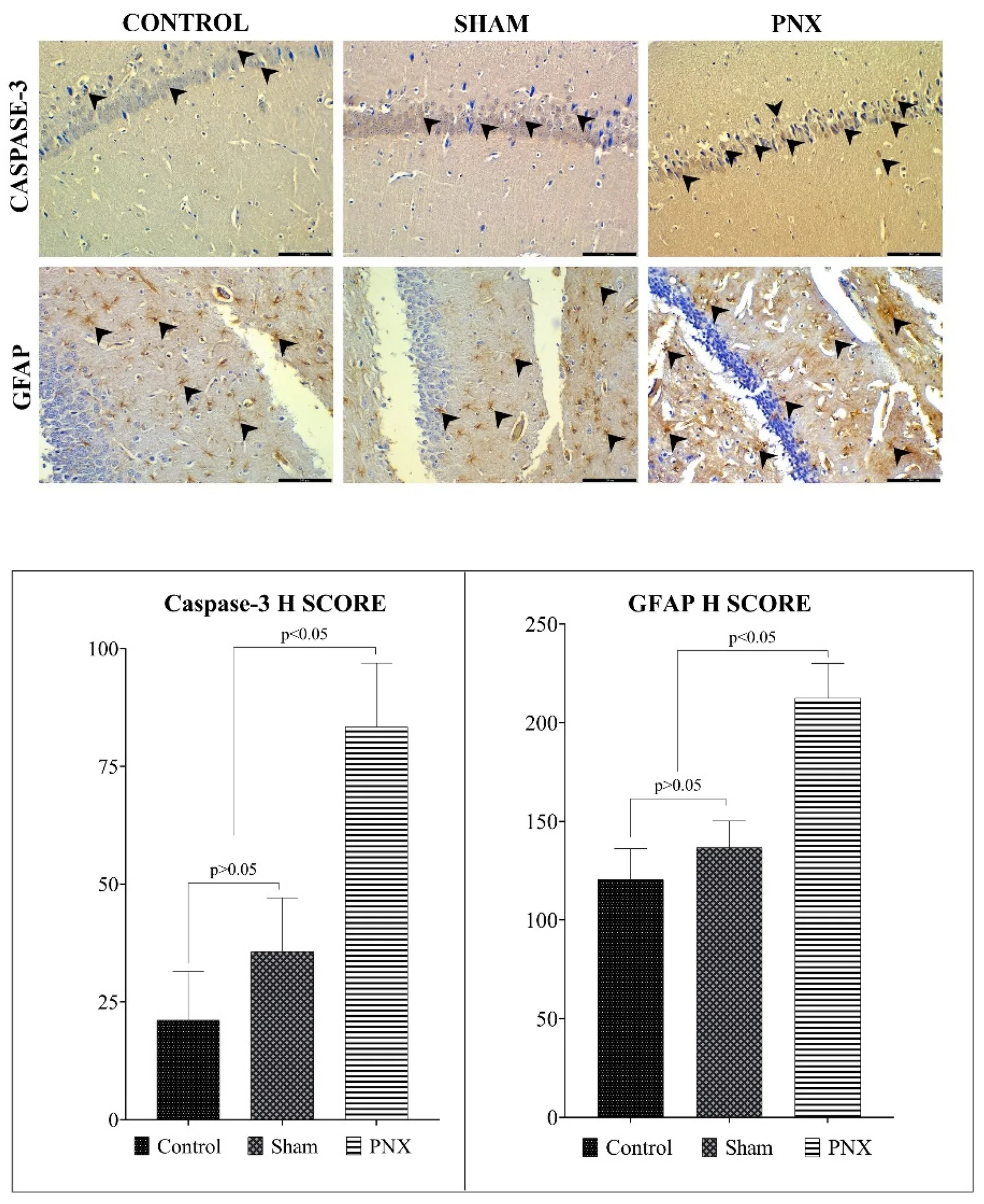
Immunohistochemical staining of caspase-3 and GFAP in hippocampal tissue of Control, Sham, and PNX groups, along with their corresponding H-score analyses. Arrowheads indicate cells presenting positive immunoreactivity. Data are expressed as mean ± SD (*n* = 10), and differences were considered statistically significant at *p* < 0.05. For caspase-3, the PNX group (83.413 ± 13.536) showed a significant increase compared with the Control (21.134 ± 10.345) and Sham (36.675 ± 11.463) groups (*p* < 0.05). Similarly, for GFAP, the PNX group (212.435 ± 17.535) was significantly higher than the Control (120.436 ± 15.753) and Sham (136.684 ± 13.546) groups (*p* < 0.05). No statistically significant differences were observed between the Control and Sham groups for either caspase-3 or GFAP H-scores (*p* > 0.05).

Immunohistochemical staining for caspase-3 revealed the highest number of immunoreactive cells in the PNX group compared with the other groups (*p* < 0.05). The Control and Sham groups had comparable staining patterns (*p* > 0.05), with statistically significant lower scores relative to the PNX group (*p* < 0.05) (Figure 4).

#### 3.2.2. GFAP Immunohistochemical Staining

Immunohistochemical detection of GFAP revealed the highest density and number of immunoreactive cells in the PNX group compared with the other groups (*p* < 0.05). The Control and Sham groups exhibited comparable staining patterns (*p* > 0.05), yet these groups demonstrated significantly lower scores in comparison to the PNX group (*p* < 0.05) (Figure 4).

### 3.3. Results of Biochemical Analyses

**Figure 5:**
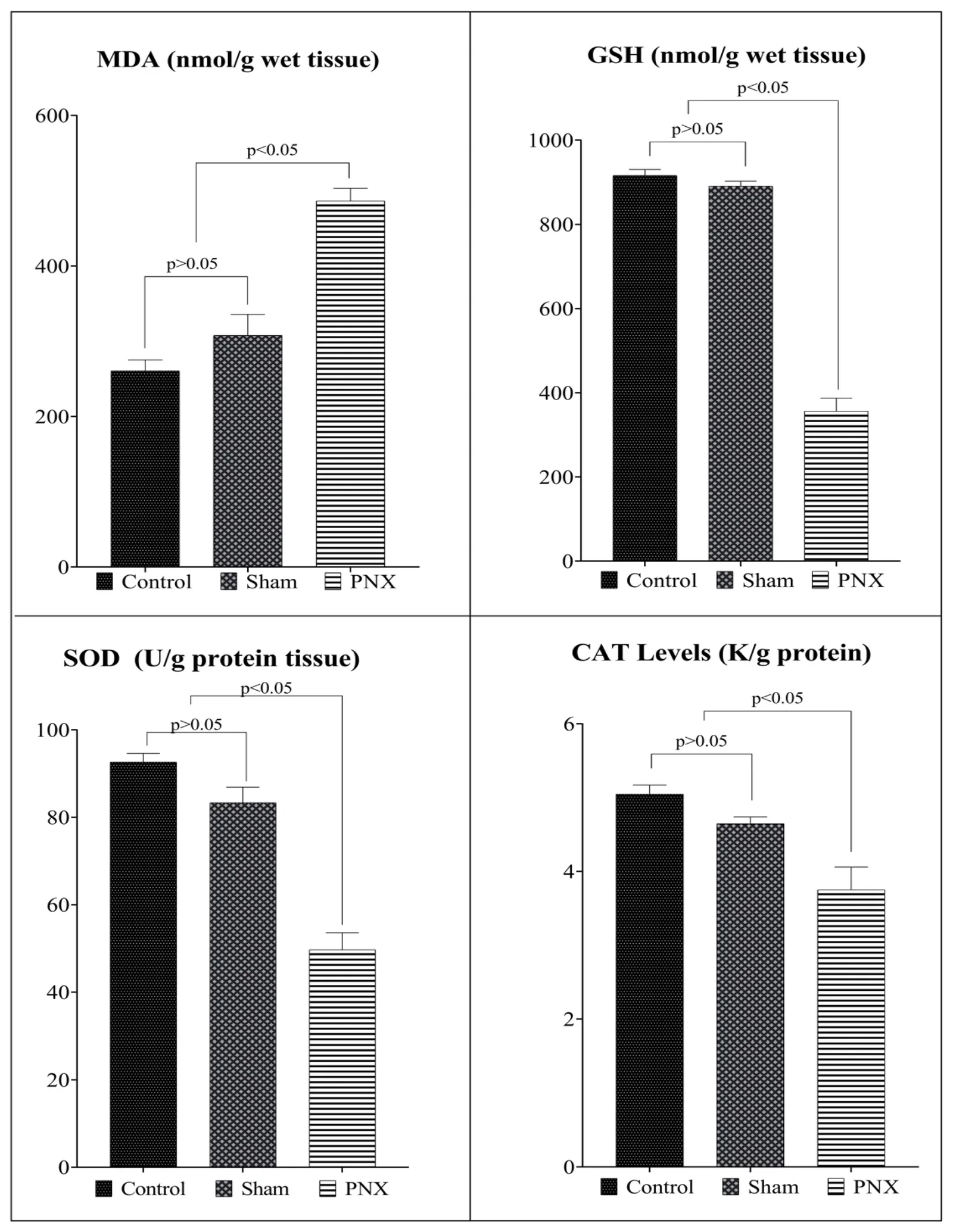
Results of hippocampus MDA, GSH, SOD, and CAT levels. Differences were considered statistically significant at *p* < 0.05. Data are expressed as mean ± SD (*n* = 10). MDA, GSH, SOD, CAT; PNX compared with Control and Sham (*p* < 0.05). No statistically significant differences were observed between the Control and Sham groups (*p* > 0.05).

**Figure 6:**
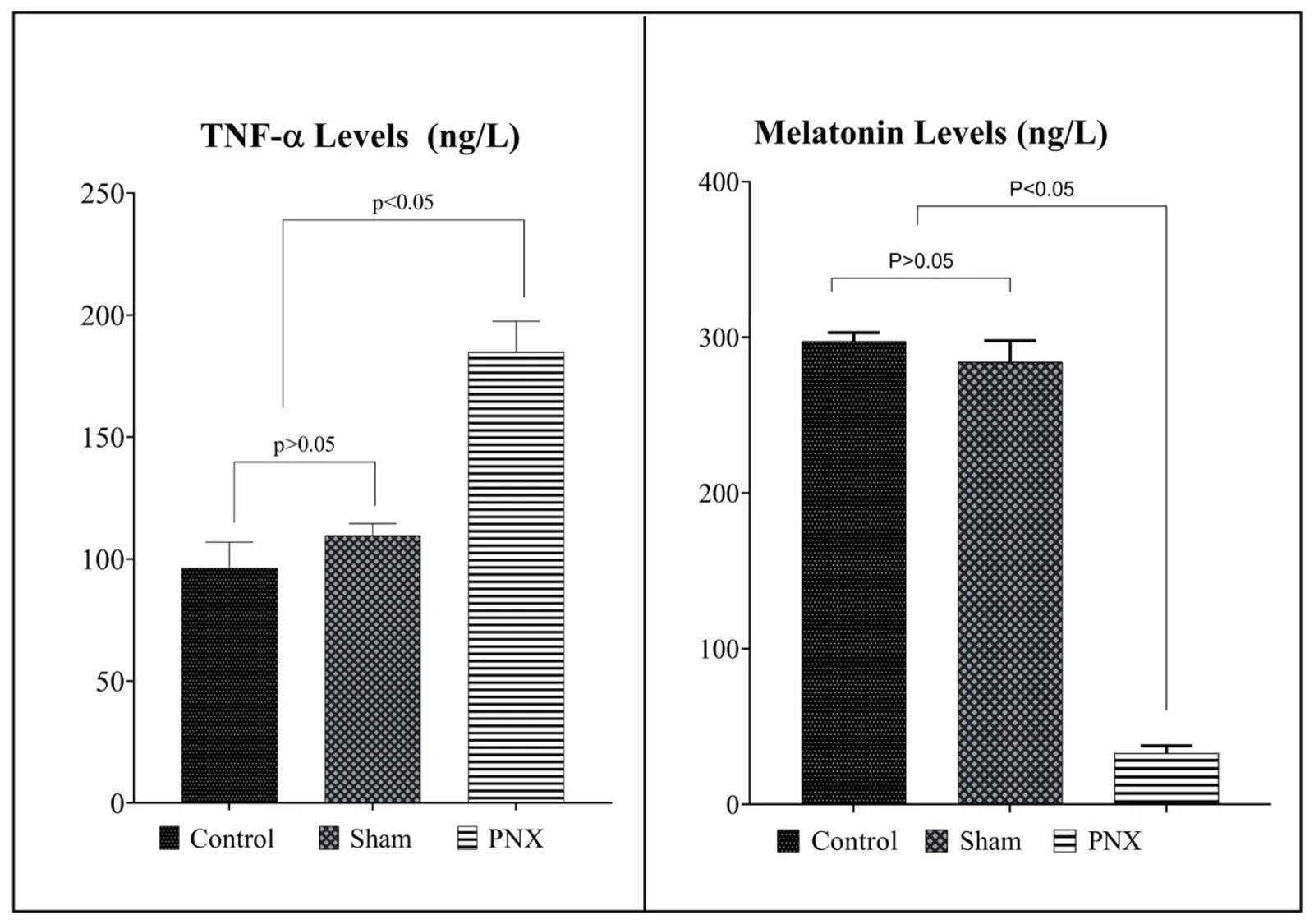
TNF-α levels in hippocampal tissue and serum melatonin levels. Data are expressed as mean ± SD (*n* = 10). Differences were considered statistically significant at *p* < 0.05. The PNX group showed significant differences compared with the Control and Sham groups (*p* < 0.05), whereas no statistically significant differences were observed between the Control and Sham groups (*p* > 0.05).

We assessed neuronal oxidative stress by quantifying the amounts of MDA, GSH, SOD, and CAT. Figure 5 encapsulates the results. The results indicated that pinealectomized rats exhibited a considerable elevation in MDA levels (PNX: 486.274 ± 16.932 nmol/g wet tissue) compared with the control (260.716 ± 14.362 nmol/g wet tissue) and sham groups (307.625 ± 28.045 nmol/g wet tissue) (*p* < 0.05), along with a significant decrease in GSH levels (PNX: 356.174 ± 31.065 nmol/g wet tissue) compared with the control (916.183 ± 14.134 nmol/g wet tissue) and sham groups (891.126 ± 11.384 nmol/g wet tissue) (*p* < 0.05), and the activities of SOD (PNX: 49.682 ± 3.902 U/g protein tissue; Control: 92.625 ± 1.994; Sham: 83.367 ± 3.546 U/g protein tissue, *p* < 0.05) and CAT (PNX: 3.751 ± 0.307 K/g protein; Control: 5.048 ± 0.123; Sham: 4.645 ± 0.094 K/g protein, *p* < 0.05) when compared to the control and Sham groups. We also assessed neuronal inflammation by quantifying TNF-α levels. According to our results, pinealectomized rats exhibited a marked elevation in TNF-α levels (PNX: 184.705 ± 12.725 ng/L) compared with the control (96.215 ± 10.723 ng/L) and sham groups (109.589 ± 4.935 ng/L) (*p* < 0.05). The results were given in Figure 6. Melatonin levels were also evaluated at the end of the study. According to our results, pinealectomized rats exhibited significantly lower melatonin levels (PNX: 32.714 ± 4.893 ng/L) compared with the control (297.386 ± 5.684 ng/L) and sham groups (284.127 ± 13.711 ng/L) (*p* < 0.05). The results are summarized in Figure 6.

## 4. Discussion

Based on accumulating evidence indicating that pinealectomy is associated with biochemical and histopathological alterations in brain tissue, we postulated that melatonin deficiency may be associated with alterations in the molecular architecture of the circadian clock, rather than acting solely through antioxidant depletion. To address this hypothesis, we focused on the core components of the molecular clock, analysing the expression of the positive regulatory arm (CLOCK and BMAL1) and the negative feedback regulators (PER1 and CRY1). Consistent with gene expression findings, pinealectomy was associated with the coordinated suppression of core circadian clock gene expression in the hippocampus. This finding suggests alterations in the circadian transcriptional network following melatonin deprivation. It is well established that pinealectomy reduces circulating melatonin levels. Given the pivotal role of melatonin in circadian regulation, molecular evaluation of core circadian clock gene expression is particularly significant. Although the relationship between melatonin signaling and circadian regulation has previously been investigated, few studies have examined simultaneously hippocampal core clock gene expression, oxidative stress, neuroinflammation, and histopathological alterations following pinealectomy.

Pinealectomy-induced melatonin deficiency has been associated with increased vulnerability to oxidative stress and inflammation, processes that are tightly linked to the dysregulation of circadian clock gene networks governing physiological homeostasis [26]. Melatonin, which is rhythmically regulated by the circadian clock, acts as an effective free radical scavenger [49].

Oxidative stress and inflammation are recognized contributors to neurodegeneration in the central nervous system [50]. In our study, the marked oxidative imbalance observed following pinealectomy coincided with a significant downregulation of core circadian clock genes, suggesting a potential link between circadian dysregulation and redox dysregulation in the hippocampus.

Moreover, as the measurements were obtained at a single time point, the dynamic oscillatory nature of circadian gene expression is not fully captured, which should be taken into account when interpreting these findings.

In addition to this unidirectional interpretation, the relationship between circadian dysregulation and oxidative stress is likely to be bidirectional. While melatonin deficiency and downregulation of core clock genes may contribute to oxidative imbalance, increased oxidative stress may in turn further impair circadian clock function by affecting redox-sensitive transcriptional mechanisms. This reciprocal interaction suggests the presence of a maladaptive feedback loop, in which circadian dysregulation and oxidative stress reinforce each other, potentially exacerbating hippocampal vulnerability following pinealectomy. Although the present study does not directly investigate this bidirectional mechanism, the combined molecular and biochemical findings support this integrative perspective.

Previous studies have shown that pinealectomy shifts the antioxidant–oxidant balance toward oxidative stress and inflammation [38,51]. Consistent with this, our study observed a significant increase in MDA levels, accompanied by marked reductions in GSH, SOD, and CAT activities, in the hippocampus of pinealectomized rats compared with the control and sham groups. These findings agree with earlier reports, confirming that pinealectomy induces a pronounced oxidative imbalance in brain tissue. Notably, the oxidative imbalance observed after pinealectomy was accompanied by a marked suppression of core circadian clock genes, suggesting an association between circadian dysregulation and impaired redox homeostasis in the hippocampus.

Melatonin has been shown to modulate pro-inflammatory cytokine production, and disruption of melatonin signaling is associated with elevated IL-6 levels [52]. Consistently, Permpoonputtana et al. demonstrated that melatonin supplementation significantly attenuated increased IL-6 protein levels in the hippocampus, highlighting its potent anti-inflammatory effects [53]. In our study, the pronounced increase in IL-6 levels following pinealectomy was accompanied by significant suppression of core circadian clock genes, suggesting that circadian dysregulation may contribute to the amplification of neuroinflammatory signaling in the hippocampus.

Taken together, the present findings suggest a close interplay between circadian dysregulation, oxidative stress, and neuroinflammation in the hippocampus following pinealectomy. Downregulation of core circadian clock genes may impair redox homeostasis and facilitate pro-inflammatory signaling. At the same time, increased oxidative stress and inflammatory mediators may further disrupt circadian regulatory mechanisms through redox-sensitive and cytokine-mediated pathways. This integrated framework indicates that these processes do not act independently but rather form a mutually reinforcing network that may contribute to hippocampal vulnerability. Although the present study does not directly establish causal relationships among these pathways, the combined molecular, biochemical, and histopathological findings support this interconnected model.

One limitation of the present study is the absence of assessments of key enzymes involved in melatonin biosynthesis, such as AANAT and ASMT/HIOMT, and of serotonin levels. A detailed evaluation of melatonin synthesis pathways could provide a deeper understanding of the mechanisms behind the observed circadian dysregulation and neuroinflammatory processes following pinealectomy.

Another limitation of this study is that an a priori power analysis was not performed to determine sample size. Although statistically significant differences were observed across multiple parameters, future studies incorporating formal power calculations would further strengthen the robustness of the findings.

An additional limitation of this study relates to species-specific differences in circadian organization. Rodents are nocturnal, whereas humans are diurnal, and therefore circadian regulatory mechanisms and their physiological outputs are not directly comparable between species. This difference may influence the interpretation of hippocampal responses to circadian dysregulation and melatonin deficiency. Although rodent models provide valuable mechanistic insights into circadian biology, caution is warranted when extrapolating these findings to human conditions.

Although sampling was performed at a fixed time point, it should be noted that circadian gene expression exhibits dynamic fluctuations throughout the day. Therefore, single time-point measurements may not fully capture the temporal profile of circadian regulation.

The lack of cell-type-specific gene expression analysis represents another limitation of this study. Future studies employing neuron- or glia-specific isolation approaches would provide more precise insight into circadian gene regulation at the cellular level.

Moreover, pinealectomy has been reported to trigger an inflammatory response and significantly increase TNF-α levels in rats [54]. Consistent with these findings, we observed marked elevations in TNF-α and IL-6 levels in pinealectomized rats compared with the control and sham groups. This inflammatory profile may be associated with oxidative stress and the activation of neuroinflammatory pathways in the context of melatonin deficiency. Notably, the inflammatory activation observed after pinealectomy suggested a potential role for circadian dysregulation in amplifying neuroinflammatory responses.

Kus et al. evaluated the effects of pinealectomy and exogenous melatonin administration on the CA1, CA2, and CA3 regions of the hippocampus in rats [55]. Our results align with theirs; nonetheless, it is noteworthy that the researchers assessed these effects in rats that had undergone pinealectomy 3 months prior, whereas this study is evaluating 30 days after pinealectomy. The researchers considered cells with pyknotic nuclei a sign of cell death; however, they did not assess established markers of apoptosis. In our work, an anti-caspase-3 antibody was employed to evaluate the cell death cascade, revealing statistically significantly higher expression levels in the pinealectomy group compared with the other two groups (*p* < 0.05). Our findings suggest an association between melatonin deficiency and apoptosis-related alterations in hippocampal cells. Unlike this and other study groups, we established that, to our knowledge, limited data exist showing that caspase-3 expression increased considerably one month post-pinealectomy compared to other groups, supporting the possibility that the pineal gland may contribute to neuroprotective mechanisms in the hippocampus.

A noteworthy and constant alteration in reactive astrocytes is the considerable elevation of GFAP, an intermediate filament protein predominantly produced by astrocytes. Increased GFAP often indicates alterations in the astrocyte cytoskeleton, encompassing hypertrophy and the elongation of processes directed towards the damage site [56]. In an Alzheimer’s disease model induced by ICV STZ injection, melatonin supplementation did not affect GFAP expression; however, GFAP expression increased in the group that did not receive melatonin [57]. Our results demonstrate that melatonin deprivation (pinealectomy) elevates GFAP expression in a manner analogous to Alzheimer’s disease produced by ICV STZ injection. These findings are consistent with the proposed neuroprotective role of melatonin on preserved brain tissue. Our research indicates that the majority of studies in the literature investigating the harmful effects of neurotoxins on the hippocampus have primarily utilized anti-GFAP and anti-caspase-3 antibodies. Multiple studies have established the neuroprotective effects of melatonin against various neurotoxins. To our knowledge, only a few studies have evaluated GFAP and caspase-3 immunoreactivity simultaneously to assess hippocampal alterations following pinealectomy, thereby negating the neuroprotective effects of melatonin. It should also be considered that gene expression analyses were performed on whole hippocampal tissue. Therefore, the observed changes may partly reflect alterations in cellular composition, particularly in relation to neuronal loss and astrocyte activation, rather than transcriptional regulation alone.

BMAL1 deficiency disrupts synaptic vesicle cycling and increases susceptibility to oxidative damage and inflammation, demonstrating that circadian control is critical for maintaining neuronal function and plasticity [58]. The findings, which emerged from a study investigating the deletion of BMAL1 alone or in combination with CLOCK in mice, demonstrated that BMAL1, acting in complex with CLOCK, regulates brain redox homeostasis and links disrupted circadian clock gene function to neurodegeneration [59]. In our study, BMAL1 and CLOCK expression were reduced in the hippocampus of pinealectomized rats. The decrease in melatonin and BMAL1 and CLOCK expressions resulting from pinealectomy is accompanied by disruption of the circadian rhythm. It has been established that alterations in the expression of BMAL1/CLOCK genes modulate the transcription of various negative feedback genes, including CRY1 and PER1 [60]. The absence of a melatonin replacement group after pinealectomy means that it is not possible to draw definitive conclusions about the cause of the observed molecular, biochemical, and histopathological changes. Nevertheless, the present findings are consistent with the proposed mechanisms and provide supportive evidence. Therefore, the present findings should be interpreted as associative rather than causal, and melatonin deficiency cannot be considered the sole determinant of the observed alterations.

Despite the limited number of studies specifically evaluating PER1 and CRY1 gene expression in pinealectomized rats, as previously mentioned, the consistency of our biochemical and histopathological findings with those of neurodegenerative diseases prompted us to investigate the expression of clock genes in these diseases. A review of studies conducted in this context reveals that PER1 and CRY1 gene expression is significantly reduced in rotenone-induced Parkinson’s disease models in mice and rats [61,62].

In this study, a reduction in PER1 and CRY1 gene expression was observed, consistent with the findings reported in the aforementioned neurodegenerative diseases. However, further research is required in this field to elucidate the underlying molecular mechanisms. Nevertheless, the present study provides preliminary evidence in this relatively underexplored area.

Conversely, it has been established that BDNF levels decline in neurodegenerative conditions accompanied by neuroinflammation and oxidative stress [22]. Several studies indicate that BDNF plays a role in maintaining normal brain function and neurophysiological processes [63,64]. The scientific literature suggests that alterations in melatonin levels can impact circulating BDNF concentrations [65]. Mashhadi and colleagues reported that melatonin replacement treatment affected hippocampal BDNF concentration, rather than serum BDNF levels. Consequently, it is imperative to ascertain BDNF levels in the hippocampus in the absence of melatonin [63]. Our investigation revealed that pinealectomy was associated with decreased BDNF mRNA expression in the hippocampus. However, since BDNF protein levels were not measured, further studies using protein-level assays such as Western blotting or ELISA are required to confirm whether these transcriptional changes translate into altered BDNF protein abundance. It should also be noted that gene expression in the present study was evaluated only at the mRNA level not only for BDNF but also for the other genes analysed (CLOCK, BMAL1, PER1, CRY1, and IL-6). Therefore, future studies including protein-level analyses will be necessary to determine whether these transcriptional changes are reflected at the functional protein level.

Accumulating evidence indicates that circadian clock genes play a direct role in regulating redox balance and neuroinflammatory signaling in the brain. The CLOCK–BMAL1 transcriptional complex controls genes involved in antioxidant defense and mitochondrial homeostasis, and disruption of this system increases neuronal tissue’s vulnerability to oxidative stress. Moreover, impairment of circadian clock function has been associated with enhanced pro-inflammatory cytokine production, linking circadian dysregulation to neuroinflammation. In this context, the downregulation of CLOCK, BMAL1, PER1, and CRY1 observed after pinealectomy may be associated with the observed oxidative imbalance and elevated IL-6 and TNF-α levels detected in the hippocampus by weakening circadian control over redox and inflammatory homeostasis [25,50,59,66,67]. However, the limited mechanistic evidence available in the literature constrained the depth of interpretation in the present study. Nevertheless, the present study may provide insight into the underlying mechanisms and pave the way for future studies in this field.

From a translational perspective, restoration of circadian signaling may represent a potential therapeutic strategy to mitigate hippocampal damage associated with melatonin deficiency. Approaches such as melatonin supplementation or chronotherapy aimed at resynchronizing circadian rhythms may help restore redox balance, attenuate neuroinflammatory responses, and preserve hippocampal cellular integrity. However, since no therapeutic intervention was tested in the present study, these implications should be considered preliminary. Future experimental studies incorporating melatonin replacement or circadian-based therapeutic interventions are needed to determine whether restoration of circadian signaling can reverse or attenuate the molecular and histopathological alterations observed after pinealectomy.

A limitation of the present study is that gene expression analysis was performed using bulk hippocampal tissue. Because pinealectomy induced neuronal degeneration and astrocytic activation, changes in tissue cellular composition may influence bulk RNA measurements. Therefore, reduced transcript levels may reflect neuronal loss or altered neuron-to-glia ratios rather than true per-cell transcriptional downregulation of circadian clock genes. Future studies using cell-type-specific approaches, such as RNAscope and single-cell or single-nucleus RNA sequencing, would help clarify whether the observed transcriptional changes occur within specific hippocampal cell populations. Another limitation of the present study is the use of a single housekeeping gene (ACTB) for normalisation in qRT-PCR analyses. Although ACTB has been widely used and shown to exhibit stable expression in hippocampal and neurodegeneration-related experimental models, using multiple reference genes could provide a more reliable normalisation method. Therefore, although our results consistently demonstrated significant changes in the expression of circadian clock genes, BDNF, and IL-6, future studies should incorporate multiple validated housekeeping genes in order to further strengthen quantitative accuracy. A further limitation of this study is that apoptosis was inferred from total caspase-3 immunoreactivity. The antibody utilised in this study is capable of detecting both pro-caspase-3 and its cleaved (active) form, thereby precluding the exclusive interpretation of the observed signal as indicative of executed apoptosis. The use of additional apoptosis-specific assays, such as cleaved caspase-3 immunostaining or TUNEL analysis, would provide more specific confirmation of apoptotic cell death. Therefore, the present findings should be interpreted as indicative of apoptosis-related activity rather than definitive evidence of apoptotic cell death. It is recommended that future studies incorporating these methods be undertaken in order to provide further validation of the apoptotic mechanisms suggested by the present findings. Despite these limitations, the present study provides an integrated evaluation of circadian clock gene expression together with oxidative stress markers, inflammatory cytokines, and histopathological alterations in the hippocampus following pinealectomy. This combined molecular, biochemical, and histological approach offers a broader framework for understanding how circadian dysregulation may influence hippocampal cellular integrity.

Future studies should focus on elucidating the causal relationships among circadian dysregulation, oxidative stress, and hippocampal neuroinflammation. In particular, experimental designs incorporating melatonin replacement or circadian-based interventions would be valuable to determine whether restoration of circadian signaling can reverse or attenuate the observed molecular and histopathological alterations. In addition, mechanistic investigations targeting redox-sensitive transcriptional pathways and cell-type-specific responses would further clarify the interplay between clock gene dysregulation and neuroinflammatory processes. The use of more specific apoptosis assays and advanced quantitative histological approaches would also strengthen the interpretation of cellular injury.

## 5. Conclusions

This study investigated the impact of pinealectomy on hippocampal molecular and cellular homeostasis by focusing on the expression of core circadian clock genes (CLOCK, BMAL1, PER1, and CRY1), as well as BDNF and IL-6, using qRT-PCR. In parallel, histopathological alterations in hippocampal architecture were evaluated by immunohistochemistry for caspase-3 and GFAP. Additionally, oxidative stress markers (MDA, GSH, SOD, and CAT) and the pro-inflammatory cytokine TNF-α were quantified to provide a comprehensive biochemical profile.

Pinealectomy was associated with coordinated downregulation of core circadian clock genes in the hippocampus, suggesting alterations in circadian molecular regulation. This circadian impairment was accompanied by a marked reduction in hippocampal BDNF expression and a concomitant increase in IL-6 levels, suggesting a shift toward a pro-inflammatory and neurodegenerative milieu.

Consistent with these molecular alterations, immunohistochemical analyses revealed significantly increased caspase-3 and GFAP immunoreactivity in pinealectomized rats, together with pronounced histopathological damage in hippocampal microarchitecture.

It should be noted that histopathological observations are primarily descriptive, and although supported by semi-quantitative H-score analysis, more advanced quantitative morphometric approaches would further strengthen these findings.

In conclusion, this study suggests that pinealectomy is associated with a disruption of the hippocampal circadian clock machinery, as evidenced by the significant downregulation of the core clock genes CLOCK, BMAL1, PER1, and CRY1. This molecular circadian impairment was consistently accompanied by increased oxidative stress, reduced antioxidant capacity, enhanced neuroinflammatory signaling, and decreased BDNF expression. Pinealectomized rats also exhibited significant histopathological alterations in the hippocampus, including increased caspase-3 immunoreactivity, suggesting a potential involvement of apoptosis-related pathways, and marked astrocytic activation. The results obtained provide evidence of the critical role of intact circadian clock gene function in maintaining redox balance and cellular integrity in the hippocampus.

## Figures and Tables

**Table 1:** The primer sequences of genes.

Primer Name	Sequences 5′ => 3′	Tm
Rat_CLOCK_F	TGCTGTCCTTACTGCTTGGT	57 °C
Rat_CLOCK_R	TGGCAAAGTGGTGATACCTGA	
Rat_BMAL1_F	TGGCCAGAGTGAATGCTTTTG	54 °C
Rat_BMAL1_R	CCTGACTGGCCTGGAACTTG	
Rat_PER1_F	GTGCTCCAGGATCCCATCTG	54 °C
Rat_PER1_R	CTCTGAGAACCGTGGCTGTT	
Rat_CRY1_F	TGCGCATTTCACACACACTG	56 °C
Rat_CRY1_R	GACAGAGGGGTTGTGCACTT	
Rat_BDNF-F	CGAGACCAAGTGTAATCCCA	59 °C
Rat_BDNF-R	TCTATCCTTATGAACCGCCA	
Rat_IL-6-F	ACAAGTCCGGAGAGGAGACT	50 °C
Rat_IL-6-R	TTGCCATTGCACAACTCTTTTC	
Rat β-actin-F	CCCGCGAGTACAACCTTCT	59 °C
Rat β-actin-R	CGTCATCCATGGCGAACT	

## Data Availability

The datasets generated and/or analysed during the current study are not publicly available due to the fact that there is no suitable database, but are available from the corresponding author on reasonable request. The authors confirm that the data supporting the findings of this study are available within the article.
