# Correction: Gurbuz Can et al. Altered Hippocampal Clock Gene Regulation Is Associated with Circadian Dysregulation of Oxidative Imbalance, Neuroinflammation, and Histopathological Damage After Pinealectomy. *Biology* 2026, *15*, 655

**DOI:** 10.3390/biology15131064

**Published:** 2026-07-03

**Authors:** Venhar Gurbuz Can, Mehmet Demir, Tansu Kusat, Feyza Basak

**Affiliations:** 1 Department of Medical Biology, Faculty of Medicine, Karabuk University, Karabuk 78050, Turkey; 2 Department of Physiology, Faculty of Medicine, Karabuk University, Karabuk 78050, Turkey; mehmetdemir@karabuk.edu.tr; 3 Department of Histology and Embryology, Faculty of Medicine, Karabuk University, Karabuk 78050, Turkey; tansukusat@karabuk.edu.tr (T.K.); feyzabasak@karabuk.edu.tr (F.B.)

## Error in Figure

In the original publication [1], there was a mistake in Figure 4 as published. During the final revision process, a requested modification to Figure 4 was inadvertently not implemented in the published version of the article. The lower graph panel was inadvertently replaced with TNF-α and melatonin graphs. The correct lower panel should display the H-score analyses of caspase-3 and GFAP, consistent with the immunohistochemical findings and the values reported in the original figure legend. There is no error in the figure legend or elsewhere in the manuscript; the issue is limited solely to Figure 4. Importantly, this correction does not affect the results, statistical analyses, interpretation, or conclusions of the article. The corrected Figure 4 appears below. The authors state that the scientific conclusions are unaffected. This correction was approved by the Academic Editor. The original publication has also been updated.

## Figures and Tables

**Figure 4:**
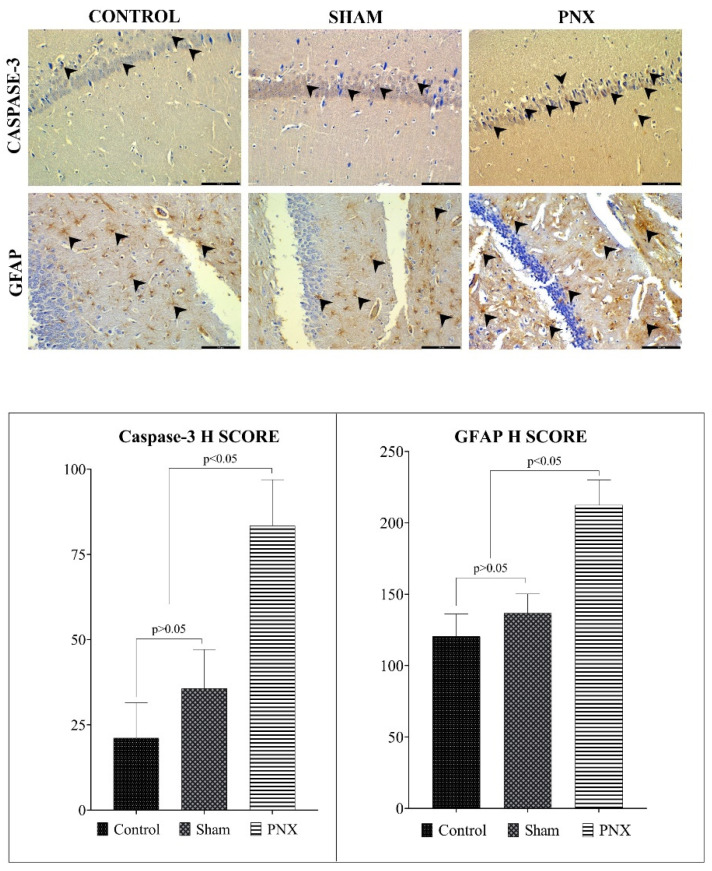
Immunohistochemical staining of caspase-3 and GFAP in hippocampal tissue of Control, Sham, and PNX groups, along with their corresponding H-score analyses. Arrowheads indicate cells presenting positive immunoreactivity. Data are expressed as mean ± SD (*n* = 10), and differences were considered statistically significant at *p* < 0.05. For caspase-3, the PNX group (83.413 ± 13.536) showed a significant increase compared with the Control (21.134 ± 10.345) and Sham (36.675 ± 11.463) groups (*p* < 0.05). Similarly, for GFAP, the PNX group (212.435 ± 17.535) was significantly higher than the Control (120.436 ± 15.753) and Sham (136.684 ± 13.546) groups (*p* < 0.05). No statistically significant differences were observed between the Control and Sham groups for either caspase-3 or GFAP H-scores (*p* > 0.05).
